# Chronic whole body vibration ameliorates hippocampal neuroinflammation, anxiety-like behavior, memory functions and motor performance in aged male rats dose dependently

**DOI:** 10.1038/s41598-022-13178-1

**Published:** 2022-05-30

**Authors:** Tamás Oroszi, Sietse F. de Boer, Csaba Nyakas, Regien G. Schoemaker, Eddy A. van der Zee

**Affiliations:** 1 Department of Neurobiology, GELIFES, University of Groningen, Groningen, The Netherlands; 2 Research Center for Molecular Exercise Science, Hungarian University of Sports Science, Budapest, Hungary; 3 Behavioral Physiology Research Laboratory, Health Science Faculty, Semmelweis Univesity, Budapest, Hungary; 4 Department of Cardiology, University Medical Center, Groningen, The Netherlands

**Keywords:** Molecular biology, Neuroscience

## Abstract

Whole body vibration (WBV) is a form of passive exercise by the stimulation of mechanical vibration platform. WBV has been extensively investigated through clinical studies with main focus on the musculoskeletal system. However, pre-clinical data in the context of behavior, memory and motor functions with aged rodents are limited. The aim of this experiment was to investigate the dose dependent effects of a five weeks long WBV intervention with an aged animal model including anxiety-related behavior, memory and motor functions, as well as markers of (neuro)inflammation. Male Wistar rats (18 months) underwent 5 or 20 min daily vibration exposure or pseudo-treatment (i.e.: being subjected to the same environmental stimuli for 5 or 20 min, but without exposure to vibrations) 5 times per week. After 5 weeks treatment, cognitive functions, anxiety-like behavior and motor performance were evaluated. Finally, brain tissue was collected for immunohistological purposes to evaluate hippocampal (neuro)inflammation. Animals with 20 min daily session of WBV showed a decrease in their anxiety-like behavior and improvement in their spatial memory. Muscle strength in the grip hanging test was only significantly improved by 5 min daily WBV treatments, whereas motor coordination in the balance beam test was not significantly altered. Microglia activation showed a significant decrease in the CA1 and Dentate gyrus subregions by both dose of WBV. In contrast, these effects were less pronounced in the CA3 and Hilus subregions, where only 5 min dose showed a significant effect on microglia activation. Our results indicate, that WBV seems to be a comparable strategy on age-related anxiety, cognitive and motor decline, as well as alleviating age-related (neuro)inflammation.

## Introduction

Aging is one of the major risk factors for various pathological conditions which entails the general decline of body functions, as well a number of structural and functional changes including loss of muscle mass and strength^1^, bone composition^2^, and change of cardiovascular and metabolic homeostatic^3^. In addition, aging impacts the central nervous system (CNS) and contributes to broad pathological changes such as decreased brain volume^4^, mitochondrial dysfunctions^5^ and increased neurodegeneration^6^. Age-related neurodegeneration is associated not only with age-related loss of neural structures, but with the progressive development of so called (neuro)inflammation^7^. During central and/or peripheral inflammatory processes, microglia, the primary immune cells of CNS become activated and produce further level of pro-inflammatory cytokines, thereby influencing and interfering with its cellular vicinity^8,9^. In addition, it has been reported that microglia show age-related increase of its threshold sensitivity for activation^10–12^. More importantly, (neuro)inflammation associated with aging has been demonstrated in preclinical literature to contribute to the progression of cognitive impairments^12–14^. It has been widely reported that aging in rodents is accompanied by impaired memory and learning functions^15–17^, increased level of anxiety-like behavioral patterns^18–20^, as well by the general decline of muscular functions^21–23^.

Physical activity has been widely acknowledged as a significant, easily available and low-cost intervention to promote body fitness at multiple levels and prevent age-related progression of neurodegenerative and other diseases^24,25^. In preclinical studies, active exercise interventions have been demonstrated to activate various protective and repair systems by releasing wide range of neurotrophic and other factors^26–28^. These factors contribute to the optimal brain homeostatic and protect the brain from pathological events including (neuro)inflammation^28–30^. Furthermore, it has been well documented in rodents that active exercise interventions improve different aspects of memory functions and domains of anxiety-like behavior^31–35^. However, certain populations for instance elderly people are not always capable to perform sufficient active exercise trainings. Thereby, there is a growing need for developing and integrating new approaches to offer alternative lifestyle and exercise interventions to enhance motor, as well cognitive abilities of these populations. Whole body vibration (WBV), type of passive stimulation by mechanical vibration platforms, may offer an alternative for active exercise trainings. In the last three decades, WBV has been demonstrated as an effective intervention to improve different domain of physical fitness, especially in the musculoskeletal system such as increased muscle strength and bone mineral density^36,37^, hormonal responses^38^, and enhanced mobility and balance have been reported^39–41^. However, other studies have shown, that similarly to active exercise, WBV may beneficially stimulate the release of various neurotransmitters^42–44^, neurotrophic factors and neurogenesis related markers^45,46^. In addition, beneficial effects of WBV on anxiety-like behavior, memory and motor functions have been reported in rodents^47,48^. More importantly, recent studies have shown that brain damage, degeneration of neurons and activation of microglia cells induced by chronic restrain test and stroke model were alleviated in adult mice after long-term vibration exposure^46,47^. However neurobiological data with aged rodents are limited, although vibration has been considered as an alternative exercise intervention for elderly populations, especially for top aged individuals^40,41^.

Therefore, the current study was designed to obtain more insight into the influence of long-term WBV on aged male rats (18–20 months). The age of 18–20 months was chosen, because rats at this age are supposed to have an early and detectable age-related progression of (neuro)inflammation, as well as deficits in learning, memory and motor performance^15–21^.

We investigated whether WBV can affect hippocampal functioning associated with neuroinflammation, anxiety-like behavioral patterns and motor performance. It has been reported that WBV can show dose-dependent effects^48^. We therefore examined the dose-dependent effects of WBV by using sessions of different duration.

## Material and methods

### Animals

Thirty male Wistar rats were used from our own breeding colony. Age of these animals were 18 months at the start of the intervention, respectively. Animals were randomly divided into two treated groups (sessions of 5- and 20-min vibration) and into a control group (n = 10 per group). Animals were housed together (2 rats per cage) under 12/12 dark–light cycle (light on at 7:00 a.m.) and the following laboratory conditions: temperature of 22 ± 2° Celsius and humidity of 50 ± 10%. Food and water were available ad libitum. One animal from the 20 min WBV treated group died before the end of the intervention. All methods were performed in accordance with the ARRIVE guidelines. Experiment was peformed in accordance with the relevant guidlines and regulations and were approved by the animal ethical commite of the University of Physical Education (TE-KEB/No3/2020).

### Whole body vibration procedure

**Figure 1:**
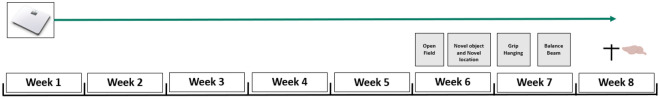
Experimental Design: Animals underwent five weeks of whole body vibration (WBV) intervention with either 5 or 20 min daily sessions, five times per week. After five weeks, a test battery was performed to evaluate anxiety-like behavior, memory functions and motor performance. Animals were terminated on week 8 and brain tissue was collected for immunohistological purposes.

A low intensity vibration platform (MarodyneLiV—Low Intensity Vibration; BTT Health GmbH; Germany) was utilized for vibration treatments. Animals underwent a vibration sessions of 5 or 20 min per day, five times per week for five consecutive weeks (Fig. 1). This platform offers a constant and low intensity vibration (frequency of 30 Hz and amplitude of 50–200 micron) with sinusoidal nature for an object weighted in the range of 20–120 kg. Thereby it was necessary to adjust the vibration platform for rats by placing a metal dumbbell (25 kg) on the top of the vibration platform. The parameters of oscillation with the experimental settings were tested and verified by additional measurements using a accelerometer (frequency: 29.6 Hz; amplitude: 0.01–0.03 mm). Animals did not receive prior habituation to the treatment procedure. Furthermore, animals were not constraint during the treatments , however, they showed slightly exited unprompted motor activity during the first 3–5 days of the intervention, but from the second week onward, they mainly remained in sitting and/or lying position. During the treatments, animals were in an empty cage with identical parameter as the home cage (36 × 18 × 23 cm) and were placed on the top of the vibration platform (i.e.: directly on the top of the metal dumbbell). The 5 and 20 min sessions were combined in the pseudo treated group and were rotated during the intervention by the same way but without actual vibration exposure. In addition, animals of two cages were treated parallel (i.e.: two cage was placed on the top of the platform) however the animals of these different cages had no social interactions with each other during the treatments. All treatment sessions were performed in the morning between 10 and 11 a.m. in a seperated experimental room. We adhered to the new reporting guidelines for WBV studies in animals ^49^.

### Test procedure

After 5 weeks of vibration treatment, two weeks long test battery was conducted including open field, novel and spatial object recognition, grip strength test and balance beam to evaluate cognitive and motor functions, as well emotionality (Fig. 1). All of these tests were conducted between 10 am and 2 pm, in a quite test room under dimmed light conditions. During the test procedures WBV treatment was given at the end of the day. In addition, animals performed two days of vibration treatment on week 8.

### Open field

Standard open field test was used to evaluate emotionality, anxiety-like behavior and unprompted motor performance induced by novel environment^50^. The dimensions of the open field test box used in this study were the followings: circular shape with the diameter of 80 cm and surrounded by a 45 cm tall wall. The test area was divided into 20 sectors by black circular and radial lines. Rats were placed in the center of the test area and were allowed to explore freely the novel environment for five minutes. Video records were taken about the procedures. The unfamiliar environment induced horizontal mobility and vertical activity (i.e.: rearing) were assessed by video analysis of the video records by ELINE software^51^. The test box was cleaned out with 70% ethanol solution and dry paper tissue between all animals. The following final outcome variables were determined: frequency of line crossings, the cumulative time spent in the center and wall sectors of area and the frequency of rearing.

### Novel and spatial object recognition

Novel and spatial object recognition test series were used to evaluate the spatial and object domains of memory functions described earlier by others^52^. This test series consists of four separated phases, all of them with the duration of 3 min and with 1 min time gap between all phases. In the first phase, the rat was placed into the test box and was allowed to explore it freely for 3 min. In the second phase, two identical objects were placed into the test box in parallel position and the rat was familiarized to these objects for 3 min. In the third phase (NLR—novel location recognition test), the two same object was placed back in diagonal position (i.e.: the original position of one of these objects was changed). In the last phase (NOR—novel object recognition), one of the familiar objects was replaced by a novel object. Two sets of objects were used and their role was randomly rotated during the procedures. Between all phases, the objects were removed from the test box and were cleaned by 70% ethanol and dry paper tissue. Animals who had no interactions with the objects (i.e.: the total exploration time was zero) were excluded at the final statistical analysis (NOR phase: 2 rats from the pseudo group and 1 rat from the 5 min session group; NLR phase: 1 rat from the pseudo treated group). Video records were taken and were analyzed by visual observation by the ELINE software. Frequency of exploration and preference time were determined as final outcome measures. Preference time was calculated by the following formula:

$$\frac{{\text{Time spent at the novel object or relocated object}}}{{\text{Total object exploration time}}}*100$$

### Balance beam

A wood beam (150 cm long and 4 cm wide) fixed in horizontal position 1 m above the surface was used to assess motor coordination by balance beam test^53^. The test procedure consisted of one familiarization day and two testing days. The home cage of animals was placed to the end of the beam and served as motivation factor. A photo cell was used to measure the walking time on the beam (i.e.: one sensor was placed to the start point while another one to the end point). Between each animal, the beam was cleaned by 70% ethanol solution and dry paper tissue.

Rats were habituated to the test environment on the first day by 3 progressive trials (from 50 cm, 100 cm and 150 cm walking distance). Rats performed two familiarization trials (from 50 and 100 cm) and three complete trials (from 150 cm) on the second and third days. The mean of the three best results from second and/or third days served as final outcome variable.

### Grip hanging test

As in case of balance beam, grip strength test was measured on three consecutive days to assess muscle performance of the fore limbs^54^. All days consisted of 3 test trials, however the first day only served as habituation day. At the start of the procedure, animals were gently picked up and supported by their trunk and allowed to try to grasp a suspended steel wire (2 mm diameter, 35 cm long, 50 cm above the surface) with their forepaws. The time until falling down was measured manually by a second researcher. Animals were rotated thought the three trials to ensure enough recovery time and avoid potential injuries induced by muscle fatigue. The test apparatus was cleaned with 70% ethanol solution and dry paper tissue after each trial. The mean of the three best results from the second and/or third days were used as final outcome measure.

### Sacrifice

Animals were terminated on week 8 (24 h after their last vibration treatment) under pentobarbital anesthesia (60 mg/kg, ip) by transcardial perfusion with saline (containing 500 u/ml heparin). Brain tissue was collected and postfixed in 4% Paraformaldehyde (PFA) solution for 4 days and stored in phosphate-buffered solution containing 0.2% sodium azide (PBSA) until the freezing procedure. Brains were cryoprotected by 30% sucrose solution and frozen by liquid nitrogen. Brains were sectionized into 25 um thick sections and stored in PBSA solution on 4 $^\circ{\rm C}$ in cold room.

### Immunohistochemistry

To visualize microglia, free floating dorsal hippocampal sections were incubated for 3 days with 1:2500 rabbit anti-IBA1 primary antibody (Wako, Neuss, Germany—Rabbit anti IBA-1) in 2% bovine serum albumin (BSA) and 0.1% Triton-X at 4 °C followed with 1:500 goat-anti rabbit secondary antibody (Jackson, Wet Grove, USA—Goat Anti Rabbit) at room temperature. Sections were incubated for 2 h with avidin–biotin peroxidase complex (Vestastain ABC kit, Vector, Burlingame, USA) and visualized with 0.075 mg/ml DAB solution. All solutions were prepared in 0.01 M phosphate-buffered solution (PBS). Through the staining procedure 0.01 PBS was used to rinse the sections between the incubation steps. Sections were mounted on glass slides and dehydrated through ethanol and xylol solutions.

### Microglia analysis

Images were taken about the subregions of the dorsal hippocampus including cornu ammonis 1 (CA1), cornu ammonis 3 (CA3), Dentate gyrus inner blade and Hilus regions by Olympus system (200 × magnification). Images were analyzed with Image-pro Plus 6.0. software (Media Cybernetics, Rockville, MD, USA). Microglia activation was calculated as described earlier by Hovens et al.^55^.

### Statistical analysis

Statistical analysis was performed by Statistica 13.2 Software. One-way ANOVA followed by Tuckey post-hoc test was used in case of all outcome variables to reveal significant interaction between the experimental groups. In addition, preference time of each individual groups was compared to the chance level by an independent sample T-test. Statistical significance was set at p < 0.05. Graphs were made by using GraphPad Prism 8.0. Software. Data are expressed as mean ± SEM.

## Results

### Open field

**Figure 2:**
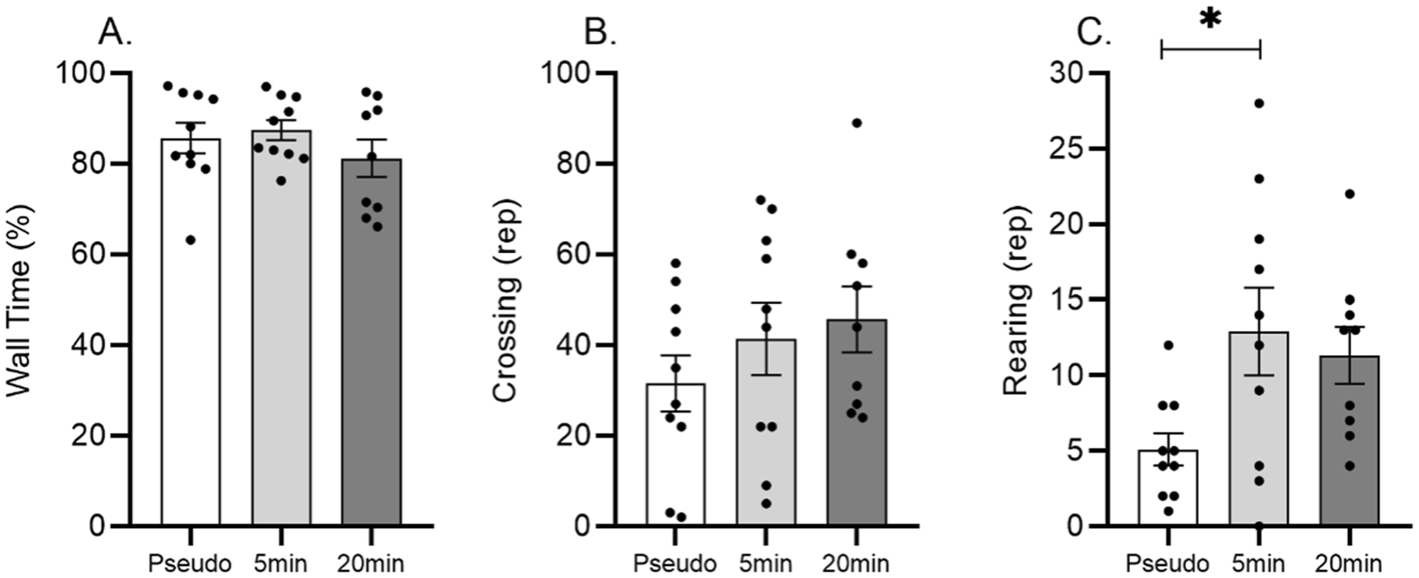
The effects of 5 and 20 min daily sessions of WBV on exploratory and anxiety-related behavior in the open field in aged male rats. The time spent at wall (panel **A**), number of crossings (panel **B**) and frequency of rearings (panel **C**). One-way ANOVA was used to reveal statistical difference between the experimental groups. Data are depicted as mean ± SEM. * indicates P < .05.

Open field test was performed to evaluate unprompted exploratory and anxiety-like behavior. Ona-way ANOVA did not show significant difference in the non-wall time/wall time (Fig. 2A) (F_(2.26)_ = 0.91; p = 0.411) as well as in the number of crossings (Fig. 2B) (F_(2.26)_ = 1.00; p = 0.37). Although a trend of decreased wall time (and increased non-wall time) and increased crossings was visible in the 20 min vibration treated groups compared to pseudo control animals.

Regarding vertical activity, significant increase (F_(2.26)_; = 3.99; p = 0.030; p_(5 min vs. pseudo)_ = 0.032) was only found in the total number of rearing in the 5 min vibrated group compared to the control group (Fig. 2C). In addition, a similar tendency of increased rearing (F_(2.26)_ = 3.99; p_(20 min vs. pseudo)_ = 0.112) was visible in the 20 min vibration group.

### Novel and spatial object recognition

**Figure 3:**
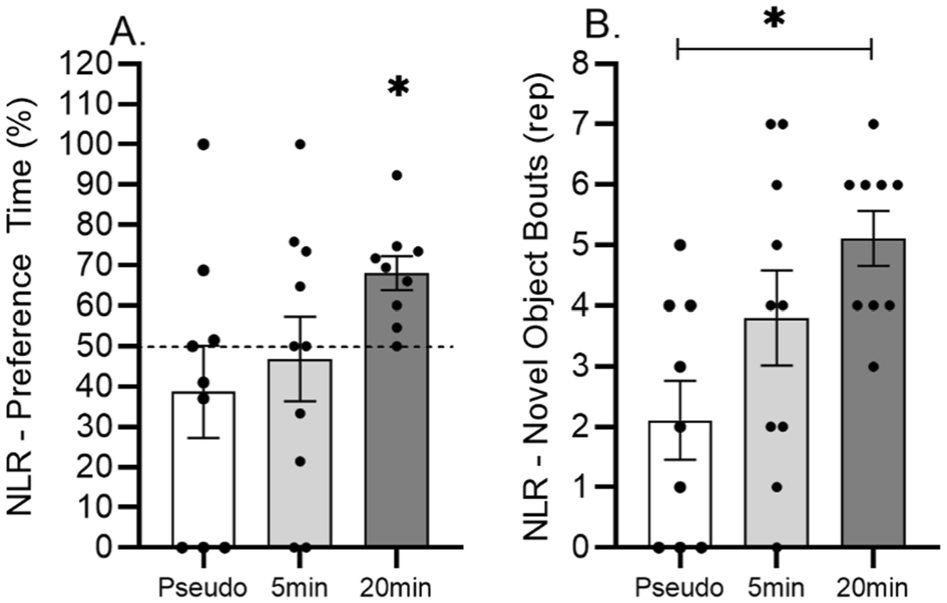
Effects of 5 and 20 min daily sessions of WBV on spatial memory in spatial object relocation test (NLR). Preference time in % (panel **A**) and frequency of novel object exploration (panel **B**). One-way ANOVA was used for reveiling the difference between the experimental groups. Further, independent T-test was used for comparing preference time to the 50% chance level (dashed line). Data are depicted as mean ± SEM. * indicates P < .05.

Novel location (NLR) and novel object recognition (NOR) tests were used to assess the spatial and object aspects of memory functions. During the NLR test, one-way ANOVA revealed a significant increase of novel object’s exploration frequency (F_(2.25)_ = 5.02; p = 0.014; p_(20 min vs. pseudo)_ = 0.011) (i.e.: number of bouts) in the 20 min vibration treated group compared to the pseudo controls (Fig. 3B). In addition, 20 min daily sessions of vibration also significantly increased the ability (p = 0.002) in the NLR test to discriminate between the familiar and novel locations of the objects compared to the chance level (Fig. 3A) (i.e.: * it reflects the significant difference compared to the chance level of 50%). This discrimination parameter was not affected in the 5 min vibration and pseudo treated groups (p = 0.772 and 0.353).

**Figure 4:**
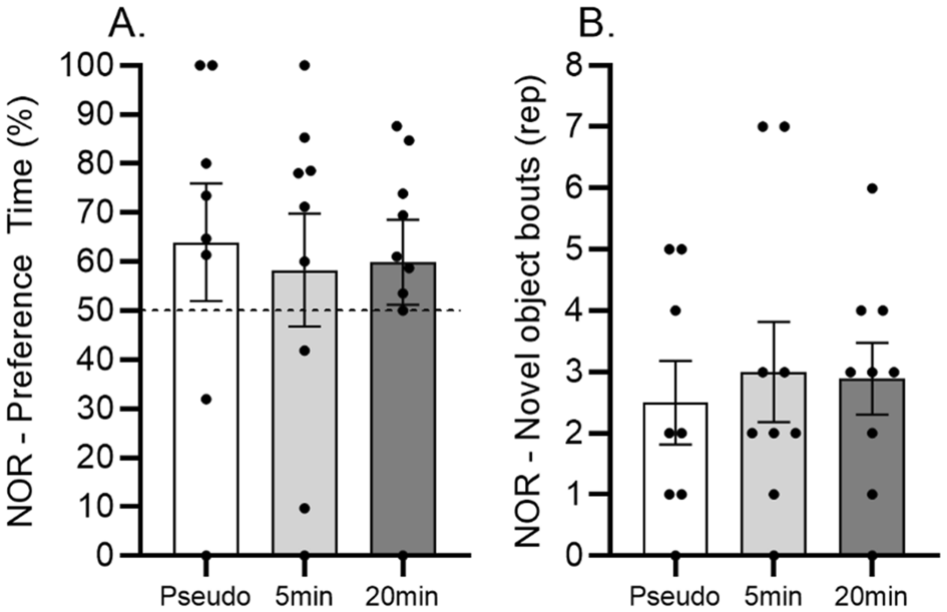
Effects of 5 and 20 min daily sessions of WBV on object memory in the novel object recognition test (NOR). Preference time in % (panel **A**) and frequency of novel object exploration (panel **B**). One-way ANOVA was used for reveiling the difference between the experimental groups. Further, independent T-test for single sample was used for comparing preference time to the 50% chance level (dashed line). Data are depicted as mean ± SEM. * indicates P < .05.

During the NOR task, no difference was found in the preference time (F_(2.23)_ = 0.071; p = 0.931) and in the frequency of exploration (F_(2.23)_ = 0.133; p = 0.875) between all groups (Fig. 4A and B). In addition, no increased preference time and frequency of exploration were explored during the familiarization phase (i.e.: familiarization phase before the NLR and NOR phases).

### Balance beam and grip hanging tests

**Figure 5:**
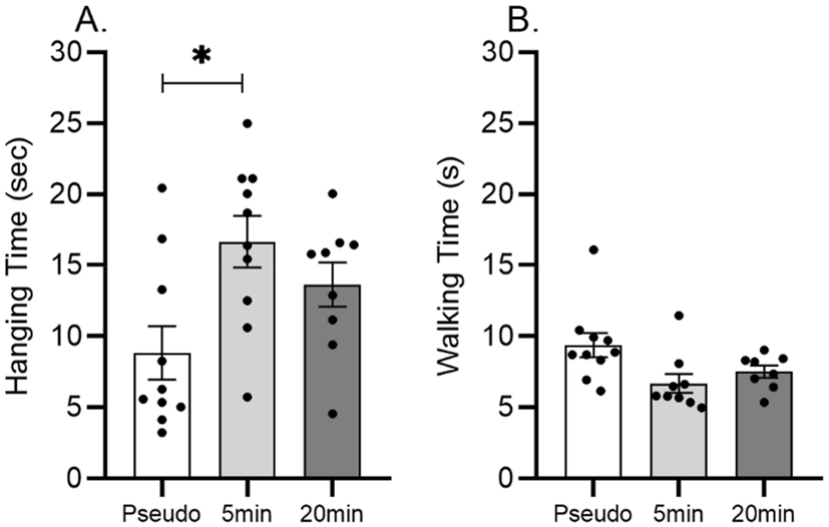
Effects of 5 and 20 min daily sessions of WBV on muscle strength in grip hanging test (panel A) and motor coordination in balance beam test (panel **B**). Data are depicted as mean ± SEM. * indicates P < .05.

Balance beam and grip hanging tests were performed for the assessment of motor coordination and muscle strength. One-way ANOVA revealed that grip strength was significantly increased by the 5 min sessions of vibration (F_(2.26)_ = 5.104; p = 0.013; p_(5 min vs. pseudo)_ = 0.010), but only a tendency was detected in the 20 min sessions of vibration group (p = 0.160) (Fig. 5A). Balance beam performance was not significantly altered (F_(2.26)_ = 0.118; p = 0.888) but a trend of decreased walking time was visible in the 5 min vibrated group (Fig. 5B).

### Microglia activation

**Figure 6:**
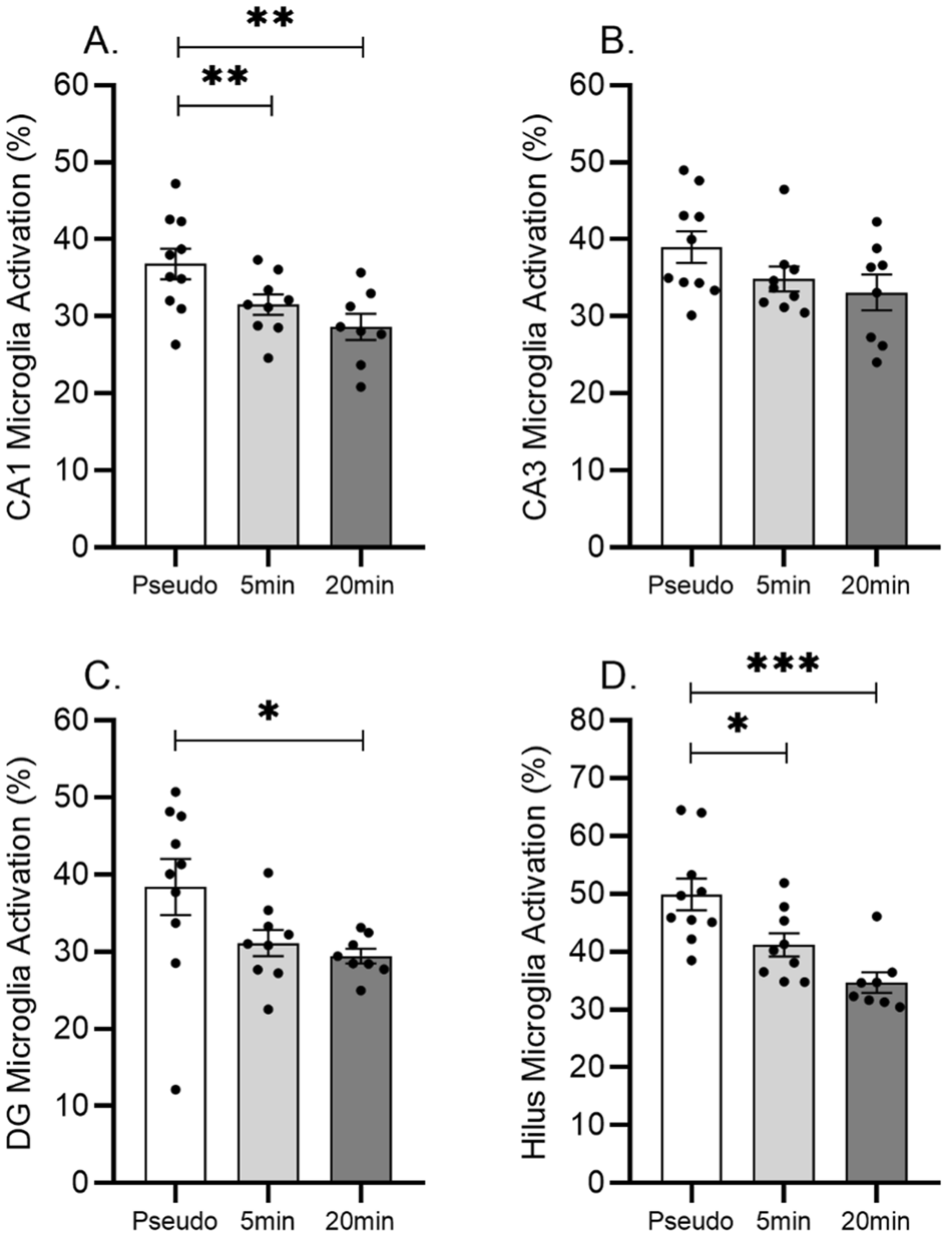
Effects of 5 and 20 min daily sessions of WBV on microglia activity in the subregions of dorsal hippocampus including CA1 (panel **A**), CA3 (panel **B**), Dentate gyrus innerblade (Panel **C**) and Hilus regions (Panel **D**). Data are depicted as mean ± SEM. * indicates P < .05; ** P < .001; *** P < .0001.

Percentage of microglia activation was determined in the subregions of the hippocampus including the CA1 (Fig. 6A), CA3 (Fig. 6B), Dentate gyrus inner blade (Fig. 6C) and Hilus (Fig. 6D) areas. A significant effect of both 5 and 20 min WBV sessions on microglia activation was only observed in the CA1 (F_(2.24)_ = 5.97; p = 0.007) and Hilus (F_(2.24)_ = 11.09; p = 0.000) regions (Fig. 6A and D). The 20 min vibration session decreased the microglia activation significantly in the Dentate gyrus inner blade (F_(2.24)_ = 3.50; p = 0.046; p_(20 min vs. pseudo)_ = 0.056), while only a same tendency (p_(5 min vs. pseudo)_ = 0.125) was visible in the 5 min vibration treated group (Fig. 6C). Microglia activation was not significantly altered in the CA3 region (F_(2.24)_ = 2.318; p = 0.120) (Fig. 6B). Total number of microglia cells did not show difference between the experimental groups (data not shown).

## Discussion

The current study investigated the dose-dependent effects of long-term vibration training with low intensity and of a sinusoidal nature on cognitive parameters, anxiety-like behavior, motor performance and neuroinflammation. The results of this experiment indicate that WBV successfully improved the motor domain of anxiety-like behavior in the open field test, spatial memory in the novel location recognition test and motor performance in the grip hanging and balance beam tests. Our data also revealed that vibration alleviates age-related markers of neuroinflammation. Overall, our results demonstrate that both daily 5 and 20 min vibration sessions for 5 weeks using the Marodyne vibration platform is able to improve motor performance, as well as brain functions and neuroinflammation in aged rats.

Clinical studies using the Marodyne platform with human patients demonstrated that it can be beneficial for musculoskeletal functions^56^. Furthermore, preclinical data with rodents suggest that WBV interventions using the Marodyne platform with low amplitude improved neuromuscular adaptation^57^, as well as contributed to enhanced glucose metabolism and accelerated muscle healing^58,59^. Taken together, low intensity vibration has successfully demonstrated, both in clinical^60,61^ and preclinical literature^62,63^, to be an alternative and low-cost treatment to mimic the beneficial effect of active exercise, especially in the musculoskeletal system. Our results corroborated these findings, and in addition show dose-dependent effects on motor functions and a positive impact of WBV on the neuroinflammatory state of the aged brain.

Open field test was conducted to evaluate emotional and anxiety-like behavior, as well as unprompted motor activity. Our results revealed that both 5 and 20 min daily sessions of WBV increased the frequency of rearing behavior, however only the 20 min daily sessions of WBV suppressed anxiety-like behavior in the open field task. Our results indicate that vibration may be beneficial to slow down the progression of age-related anxiety and motor decline. It has been well investigated that aging is accompanied by an increase of anxiety-like indices (decreased locomotion and rearing behavior, increased immobility time, more spent time in the outer zone) and the decrease of anti-anxiety indices (less spent time in the inner zone) in the open field test^18–20^. In addition, indices like horizontal mobility and especially vertical activity (rearing behavior) represent a high level of motor coordination, as well as muscle strength of the hind limbs; the age-dependent overall decline of these motor domains have also been reported^18,20^.

In general, these findings are comparable with our previous study that reported that 10 min daily sessions of WBV in rats at the age of 18–20 months is able to alleviate anxiety-like behavior and improve motor activity in the open field test^64^. Interestingly, another study^47^ showed that vibration with the frequency of 30 Hz, amplitude 4.5 mm and duration of 30 min per day has been demonstrated to alleviate anxiety-like behavior induced by restrain stress model. Taken together these results, it seems that frequency instead of amplitude may be a crucial factor to obtain the beneficial effects on rodent’s behavior with the notion that the efficiency of WBV on the different domains measured in the open field such as anxiety-related markers or unprompted motor activity seems dose-dependent.

It has been widely reported that aging in rodents is accompanied by learning and memory impairments^15–17^. Spatial memory seems to show a more pronounced and accelerated age-related decline compared to object discrimination memory^65,66^. Regarding the NLR test, the 20 min sessions of WBV group showed increased capability to distinguish the position of the relocated object in contrast to the 5 min sessions of WBV and pseudo treated animals, while the novel object discrimination index was not affected by vibration treatment. In addition, in previous study of our research group, the 10 min daily sessions of WBV was also able to improve this type of memory in aged rats^64^. Another study with young-adult mice with a similar methodical approach showed that vibration improved the performance in NOR test whereas the NLR test was not influenced^48^. These differences make clear that although WBV affects the brain positively, additional research is needed to understand whether we encountered species-specific or age-specific differences. Also, the method of WBV application (frequency and/or amplitude and/or duration) might be a critical component.

The hippocampus has been considered as a very specific brain region with a high degree of neuroplasticity, but also with a high level of vulnerability to detrimental conditions such as brain damage, chronic stress and aging leading to neuroinflammation and/or cognitive deficits^67,68^. Increasing evidence suggests that alterations in neuroimmune signaling and neuroinflammatory pathways such as increased microglia activation in the hippocampus tend to be a hallmark of the normal, non-pathological aging process^69^. The used IBA1 staining was performed to assess microglia activation in subregions of hippocampus. Both 5 and 20 min sessions of WBV decreased microglia activation, and this effect was more pronounced in the CA1, Hilus and Dentate gyrus inner blade regions. The CA3 area was less affected, but seems to be affected in the same direction, indicating that microglial activation is reduced by WBV in the entire hippocampus. This seems to be in line with the way vibrational signals reach the hippocampus. Activation of vibration-sensitive muscle and skin receptors are critical in transferring the stimuli to the cerebral cortical regions including the motor and sensory areas^70,71^. The hippocampus receives inputs from these cortical, as well other subcortical areas; these inputs reach the hippocampus through the entorhinal cortex which directly projects via the perforant paths to the cells of Dentate gyrus and CA1 areas, and more indirectly from the Dentate gyrus to the CA3 area via the mossy fibers^72^. Hippocampal responses to WBV have been reported before. One study in young adult Sprague–Dawley rats showed that WBV (30 Hz and 4.5 mm amplitude, 30 min/day, 6 days/week for 8 weeks) was able to alleviate the level of microglia-activation induced by severe immobility stress^47^. Furthermore, vibration protocols characterized by variable terms of exposure time, frequency and recovery time (multiple series of 2–3 min long vibration exposure with 1–3 min recovery time and 45 or 90 Hz of frequency) revealed beneficial electrophysiological properties of the mouse hippocampus and muscle plasticity in the early onset of aging process^73^.

Traditionally, the motor component of WBV is one of its most investigated domains. WBV has been broadly considered as an effective alternative to stimulate muscle tissue and to improve motor performance in humans^71^. In young and old rodents, WBV also improves neuromuscular functions and contributes to accelerated muscle healing^57^ and enhanced ex-vivo isometric muscle force production^59^. Likewise, WBV improves motor coordination in the balance beam test in young mice^48^. To the best of our knowledge, the beneficial effects of WBV on direct assessment of muscle strength with the grip hanging test has been reported only in middle-aged mice (15 months)^74^. We have conducted a study with the same methodical approach^64^, but with 10 min daily sessions of WBV and found increased muscle strength in the grip hanging test in 18 months old male and female rats. The decline of motor performance in aging rodents is well known. An early onset of a decline in motor tasks such as the balance beam in rats was described at the age of 15–20 months, and the decline becomes even more evident at more advanced age^75^. In the current experiment, muscle strength and motor coordination were improved in the aged rats by both 5 and 20 min sessions of WBV. However, the shorter, 5 min long session, seems to be more effective in this context. Hence, our results confirmed the efficiency of WBV in case of motor performance and data suggest that only a short time exposure of WBV may already be suitable to improve muscle performance.

It is important to emphasize that active exercise has been widely reported to improve spatial memory and learning functions in various task such as the Morris Water Maze^31^, as well the spatial object relocation test^76^. In addition, beneficial effects on various active exercise interventions have also been reported to alleviate anxiety-like behavior and improve unprompted motor activity in open field task^32–34^. Also, the protective and repair effects of active exercise interventions on the molecular and cellular level along the aging process are known, including release of growth factors^26–28^ and the alleviation of neuroinflammation^28–30^.

In general, in consideration with the literature related to active exercise interventions, our findings suggest that WBV seems to mimic the beneficial effects of active exercise interventions and could be an alternative type of exercise to improve memory functions, anxiety-like behavior and to alleviate neuroinflammation associated with aging.

## Conclusion

In conclusion, our research demonstrated that low intensity vibration with 5 and 20 min daily sessions can (1) prevent anxiety-like behavior and memory decline, (2) inhibit pathological changes in microglia related to memory decline and (neuro)inflammation, and (3) improve motor performance in 18 months old rats. Data suggest that vibration shows dose-dependent effects and acts differently in motor and cognitive domains. This is important to know as it indicates that depending on the aim of a WBV intervention, the session duration is an important variable. Although more specific and detailed mechanisms, such as molecular pathways and treatment parameters of WBV need to be further investigated, several other key aspects of the WBV protocol may also contribute to its beneficial effects during aging, as outlined in Oroszi et al., 2021^77^. Our current work provides new insights for optimally applying WBV in both preclinical and clinical studies. We hope that in the future WBV can be used as a passive exercise therapy to diminish the burden of age-related health issues and other diseases, including brain-related disorders.

## Supplementary Information

Supplementary Tables.

## Data Availability

All data generated or analysed during this study are included in this published article [and its supplementary information files].
