# Supplementary material for: Chronic whole body vibration ameliorates hippocampal neuroinflammation, anxiety-like behavior, memory functions and motor performance in aged male rats dose dependently
Source: Sci Rep. 2022 May 30;12:9020. doi: 10.1038/s41598-022-13178-1 (PMC9151803; doi:10.1038/s41598-022-13178-1)
Supplement: Supplementary file 1 — Supplementary Tables. [file 41598_2022_13178_MOESM1_ESM.pdf]

Long-term whole body vibration with a nature of low intensity ameliorates age-related neuroinflammation and improves hippocampal functioning, depression-like behavior and motor performance in aged male rats

Tamás Oroszi[1,2*], Sietse F. de Boer[1], Csaba Nyakas[2,4], Regien G. Schoemaker[1,3], Eddy A. van der Zee[1]

[1]: Department of Neurobiology, GELIFES, University of Groningen, the Netherlands
[2]: Research Center for Molecular Exercise Science, Hungarian University of Sports Science, Budapest, Hungary
[3]: Behavioral Physiology Research Laboratory, Health Science Faculty, Semmelweis Univesity, Budapest, Hungary
[4]: University Medical Center Groningen, the Netherlands

*** Correspondence:**
Corresponding Author: Tamás Oroszi
Email: oroszitomi91@gmail.com and/or t.oroszi@rug.nl

Supplementary table 1

Variable		N	Mean	Median	Min	Max	Std.Dev	SEM
OF Wall Time	ALL	29	84.88966	83.50000	63.20000	97.20000	10.14771	1.884382
	Pseudo	10	85.65000	85.10000	63.20000	97.20000	10.62787	3.360828
	5 min	10	87.42000	86.50000	76.30000	97.00000	7.08861	2.241616
	20min	9	81.2333	81.6000	66.1000	95.9000	12.37134	4.123779
OF Non-Wall TIme	ALL	29	15.11034	16.50000	2.80000	36.80000	10.14771	1.884382
	Pseudo	10	14.35000	14.90000	2.80000	36.80000	10.62787	3.360828
	5 min	10	12.58000	13.50000	3.00000	23.70000	7.08861	2.241616
	20 min	9	18.76667	18.40000	4.10000	33.90000	12.37134	4.123779
OF Crossing	ALL	29	39.34483	43.00000	2.00000	89.00000	22.30995	4.142854
	Pseudo	10	31.60000	31.00000	2.00000	58.00000	19.64801	6.213247
	5 min	10	41.40000	46.00000	5.00000	72.00000	25.18465	7.964086
	20 min	9	45.66667	44.00000	24.00000	89.00000	21.65641	7.218803
OF Rearing Number	ALL	29	9.72414	8.00000	0.00000	28.00000	7.16587	1.330669
	Pseudo	10	5.10000	4.50000	1.00000	12.00000	3.38132	1.069268
	5 min	10	12.90000	13.00000	0.00000	28.00000	9.09762	2.876920
	20 min	9	11.33333	13.00000	4.00000	22.00000	5.61249	1.870829
NOR Preference	ALL	26	60.58099	67.07516	0.00	100.0000	30.38566	5.959118
	Pseudo	8	63.94134	69.09993	0.00	100.0000	33.94882	12.00272
	5 min	9	58.29771	71.20000	0.00	100.0000	34.40586	11.46862
	20 min	9	59.87729	61.00000	0.00000	87.64045	25.98733	8.662444
NOR Novel Bouts	ALL	26	2.80769	2.50000	0.00	7.0000	2.00038	0.392308
	Pseudo	8	2.50000	2.00000	0.00	5.0000	1.92725	0.68139
	5 min	9	3.00000	2.00000	0.00	7.0000	2.44949	0.81650
	20 min	9	2.88889	3.00000	0.00000	6.00000	1.76383	0.587945
NOR Familiar Bouts	ALL	26	2.92308	2.50000	0.00	7.0000	2.01838	0.395836
	Pseudo	8	1.62500	1.50000	0.00	4.0000	1.40789	0.49776
	5 min	9	3.22222	4.00000	0.00	7.0000	2.16667	0.72222
	20 min	9	3.77778	5.00000	1.00000	6.00000	1.92209	0.640698
NLR Preference Time	ALL	28	51.05613	53.10084	0.00	100.0000	30.29487	5.725192
	Pseudo	9	38.71598	41.05263	0.00	100.0000	34.37851	11.45950
	5 min	10	46.87295	50.00000	0.00	100.0000	33.21443	10.50333
	20 min	9	68.04427	69.44444	50.00000	92.30769	12.51654	4.172179
NLR Novel Bouts	ALL	28	3.67857	4.00000	0.00	7.0000	2.29417	0.433558
	Pseudo	9	2.11111	2.00000	0.00	5.0000	1.96497	0.65499
	5 min	10	3.80000	4.00000	0.00	7.0000	2.48551	0.78599
	20 min	9	5.11111	6.00000	3.00000	7.00000	1.36423	0.454742
NLR Familiar Bouts	ALL	28	2.50000	3.00000	0.00	6.0000	1.62161	0.306456
	Pseudo	9	2.22222	2.00000	0.00	4.0000	1.56347	0.52116
	5 min	10	2.10000	2.50000	0.00	4.0000	1.37032	0.43333
	20 min	9	3.22222	3.00000	1.00000	6.00000	1.85592	0.618640
Grip	ALL	29	13.03080	13.28333	3.243333	24.99333	6.266543	1.163668
	Pseudo	10	8.849667	5.941667	3.243333	20.44000	5.929593	1.875102
	5 min	10	16.66133	17.53833	5.736667	24.99333	5.765267	1.823137
	20 min	9	13.64259	15.79667	4.570000	20.03333	4.663249	1.554416
BalanceBeam	ALL	29	9.22828	8.21000	4.996667	37.62000	6.079507	1.128936
	Pseudo	10	9.392667	8.800000	6.156667	16.07667	2.684975	0.849064
	5 min	10	9.79167	6.14833	4.996667	37.62000	9.958442	3.149136
	20 min	9	8.41963	8.21000	5.363333	15.59000	2.918583	0.972861
CA1 Activation	ALL	27	32.610	32.027	20.843	47.210	6.0496	1.1642
	Pseudo	10	36.813	36.555	26.343	47.210	6.2350	1.9717
	5 min	9	31.505	31.544	24.560	37.336	3.9288	1.3096
	20 min	8	28.601	28.340	20.843	35.677	4.8102	1.7007
CA3 Activation	ALL	27	35.856	34.617	24.029	48.980	6.3194	1.2162
	Pseudo	10	38.978	37.471	30.102	48.980	6.4680	2.0454
	5 min	9	34.852	33.653	30.463	46.500	4.8638	1.6213

	**20 min**	8	33.082	34.718	24.029	42.293	6.595	2.3316
**DGI Activation**	**ALL**	27	33.326	32.202	12.1049	50.741	8.466	1.6292
	**Pseudo**	10	38.397	40.691	12.1049	50.741	11.475	3.6286
	**5 min**	9	31.148	31.016	22.515	40.228	5.0993	1.6998
	**20 min**	8	29.438	28.964	24.987	33.104	2.6599	0.9404
**Hilus Activation**	**ALL**	27	42.491	41.307	30.4162	64.496	9.1836	1.7674
	**Pseudo**	10	49.912	47.800	38.4947	64.496	8.6466	2.7343
	**5 min**	9	41.184	40.204	34.7687	51.871	6.0079	2.0026
	**20 min**	8	34.684	33.465	30.416	46.070	5.0366	1.7807

Supplementary table 2

Variable	Test	F	p	Observed Power	Post-Hoc
**Center Time**	One-way-ANOVA	0.51681	0.602420	0.125827	
**Non-Wall Time**	One-way-ANOVA	0.91773	0.411977	0.191166	
**Center Bouts**	One-way-ANOVA	0.36546	0.697388	0.102404	
**Crossing**	One-way-ANOVA	1.00680	0.379190	0.206134	
**Rearing**	One-way-ANOVA	3.99519	0.030692	0.662709	Pseudo vs. 5min = 0.03265 Pseudo vs. 20min= 0.11251 5min vs. 20 min = 0.8602
**NLR Preference**	One-way-ANOVA	2.50936	0.101590	0.455930	
**NLR Novel**	One-way-ANOVA	5.02198	0.014675	0.765232	Pseudo vs. 5min = 0.1821 Pseudo vs. 20min= 0.01112 5min vs. 20 min = 0.34775
**NLR Familiar**	One-way-ANOVA	1.36478	0.273818	0.266041	
**NOR Preference**	One-way-ANOVA	0.07104	0.931628	0.059509	
**NOR Preference**	One-way-ANOVA	0.13369	0.875539	0.068119	
**NOR Familiar**	One-way-ANOVA	2.96212	0.071673	0.520006	
**NOR Preference vs. chance level**	Independent T-test	C = 0.283	5min: 0.48997	20min: 0.287170	
**NLR Preference vs. chance level**	Independent T-test	C: 0.353618	5min: 0.772675	20min: 0.002529	
**Grip Hanging**	One-way-ANOVA	5.1041	0.013495	0.774439	Pseudo vs. 5min = 0.0105 Pseudo vs. 20min= 0.1609 5min vs. 20 min = .4683
**Balance Beam**	One-way-ANOVA	0.11826	0.888940	0.066219	
**CA1 Activation**	One-way-ANOVA	5.972	0.007851	0.835311	Pseudo vs. 5 min= 0.0083 Pseudo vs. 20min= 0.007 5min vs. 20min= 0.487
**CA3 Activation**	One-way-ANOVA	2.3181	0.120100	0.423663	
**DGI Activation**	One-way-ANOVA	3.5006	0.046343	0.596886	Pseudo vs. 5 min= 0.1255 Pseudo vs. 20min= 0.0569

					5min vs. 20min= 0.8932
**Hilus Activation**	One-way-ANOVA	11.0984	0.000387	0.982952	Pseudo vs. 5 min= 0.0286<br>Pseudo vs. 20min= 0.0003<br>5min vs. 20min= 0.1490
